# Myositis ossificans in the pediatric population: a systematic scoping review

**DOI:** 10.3389/fped.2023.1295212

**Published:** 2023-12-15

**Authors:** Ibrahim Cherry, Marion Mutschler, Eleftheria Samara, Sophie Merckaert, Pierre-Yves Zambelli, Benjamin Tschopp

**Affiliations:** Department of Pediatric Orthopedic Surgery, Centre Hospitalier Universitaire Vaudois, Lausanne, Switzerland

**Keywords:** biopsy, child, diagnosis, imaging, myositis ossificans

## Abstract

**Introduction:**

Circumscribed or pseudomalignant myositis ossificans (MO) is a rare and benign condition characterized by heterotopic bone formation in soft tissues. The clinical presentation of MO, imaging investigations, histological findings, and treatment strategies are unclear, especially in the pediatric population.

**Materials and methods:**

A literature search was conducted in PubMed, Scopus, and Google Scholar electronic databases to identify original articles and reviews in English or French of traumatic and non-traumatic MO. Studies were selected by 2 independent reviewers following the PRISMA recommendation and descriptive data were extracted. We harvest in each case the sex, age at diagnosis, location, presence of initial trauma, pre-emptive diagnosis, modalities of imagery used, realized biopsy, treatment performed, and type of follow-up.

**Results:**

Sixty pediatric cases of MO were identified between 2002 and 2023. Twenty-three patients (38.3%) were diagnosed with idiopathic/pseudomalignant and 37 patients (61.7%) with circumscribed. The mean age at diagnosis was 9.5 years (range 0.2–17 years), with a male-to-female ratio of 1:1. The initial pre-emptive diagnosis was neoplasia in 13 patients (21.7%). The biopsy was percutaneous in 9 patients (15%) and incisional in 7 patients (11.7%). Histological analysis was achieved in 35 cases (57%). Surgical excision was the first line treatment in 46.7% of patients, and non-surgical in the remaining patients. The follow-up strategy was clinical in 16 patients (26.7%) or based on imaging investigation in 23 patients (38.3%).

**Discussion:**

Although MO in children is described as a rare pathology, identifying the benignity of the condition is essential to avoid unnecessary invasive treatment and to avoid delaying the treatment of a potentially life-threatening entity. It seems that there is no consensus established concerning the proper imaging for diagnosis. Clinicians should acknowledge that the absence of a triggering trauma tends to direct the investigation and the management toward a surgical attitude. Conservative management is key, however, surgical excision can be proposed on matured lesions on a case-by-case basis. The absence of recurrence is not excluded. Therefore, a close clinical follow-up is suggested for all cases. The true benefit of a radiological is questioned in a question known to be self-resolving.

## Introduction

Historically, the term MO has been used to describe a broad spectrum of processes ranging from benign solitary lesions to progressive congenital syndromes (e.g., MO progressiva). The literal definition of the MO was challenged since 1868 which was first described by von Dusch as a benign, self-limiting ossifying tumor occurring within skeletal muscle. The most current accepted definition of the term is a self-limiting, benign ossifying lesion that can affect any type of soft tissue, including subcutaneous fat, tendons, and nerves. It is mostly found in skeletal muscle as a solitary lesion (1). The pathophysiology of MO formation is incompletely understood (2, 3). The pathophysiologic hypothesis seems to show that it occurs through inappropriate differentiation of fibroblasts into osteogenic cells (4). Several triggering factors for this differentiation have been described such as trauma and muscle overuse.

Fundamentally, three subgroups of MO may be proposed based on etiology: MO progressiva (severe generalized form); pseudomalignant MO (without a history of trauma); circumscribed MO (related to direct trauma) (5, 6). MO progressive has been excluded from the scope of this article for its distinct clinical features and management. MO progressiva typically presents as a more severe and generalized form of myositis ossificans, affecting a broader range of muscles and requires different treatment approaches compared to the circumscribed and pseudomalignant subtypes.

Circumscribed and pseudomalignant MO are most common in men aged from 20 to 30 years old (7). They commonly involve the large muscle groups in the extremities such as the quadriceps femoris, brachialis muscle, thigh adductors, and deltoid muscle (8). The early onset involves a firm and tender mass felt within the soft tissue. Restriction of motion of the adjacent joint is typically associated. Non-traumatic and traumatic MO are usually characterized by a period of painful active growth followed by spontaneous clinical improvement and resolution. Initially, the lesion may be confused with a growing malignant tumor or an infection such as abscess. The differential should also consider granulomatous infections, sarcomas, extraosseous osteochondroma, desmoid tumors, nodular fasciitis and calcifying aponeurotic fibroma.

In the pediatric population the rarity of this pathology, variability in terminology, clinical presentation, imaging characteristics, and histopathology at times makes the diagnosis of MO challenging. Not rarely these pathologies are initially misleading as malignancies or chronic osteoarticular infections which leads to an over-treatment sometimes invasive and generates a lot of anxiety within the patient and his family.

This study aims to perform a comprehensive review of MO in the pediatric population over the last 20 years. Additionally, the demographic data and management strategy between pseudomalignant MO (without a history of trauma); circumscribed MO (related to direct trauma) groups were confronted.

## Materials and methods

### Search strategy and selection criteria

Following the recommendations of the “Preferred Reporting Items for Systematic Reviews and Meta-Analyses” (PRISMA) statement, a systematic review of the literature was performed with the following electronic databases: PubMed, Scopus, and Google Scholar. Keywords and index terms (MeSH headings) used were “MO” and “child”.

There were no language restrictions. We manually reviewed the reference lists of identified studies for further inclusions. Eligibility was independently assessed by two authors (IC and MM), and differences were resolved with the help of a third author (BT), and consensus obtained.

### Study selection

The inclusion criteria for our studies were:

- Patients under 18 years old
- Patients diagnosed with traumatic (circumscribed) and non-traumatic (pseudomalignant) MO
- Case studies published between 2002 and 2022.

The exclusion criteria were:

- Cases of progressive generalized ossifying myositis
- Follow-up period of less than 6 months.

### Data collection process

Eligibility was independently assessed by 2 authors (MM and IC), and differences were resolved with the help of a third author (BT) and consensus obtained. Full-text manuscripts were reviewed against specific inclusion criteria.

Two authors (MM and IC) independently extracted the following data: demographic characteristics (sex and age), localization of the lesion, initial pre-emptive diagnosis, imagery investigations, the need and the type of biopsy (percutaneous vs. open biopsy), treatment (conservative vs. surgical), follow-up monitoring strategy (clinical vs. radiological).

## Results

**Figure 1:**
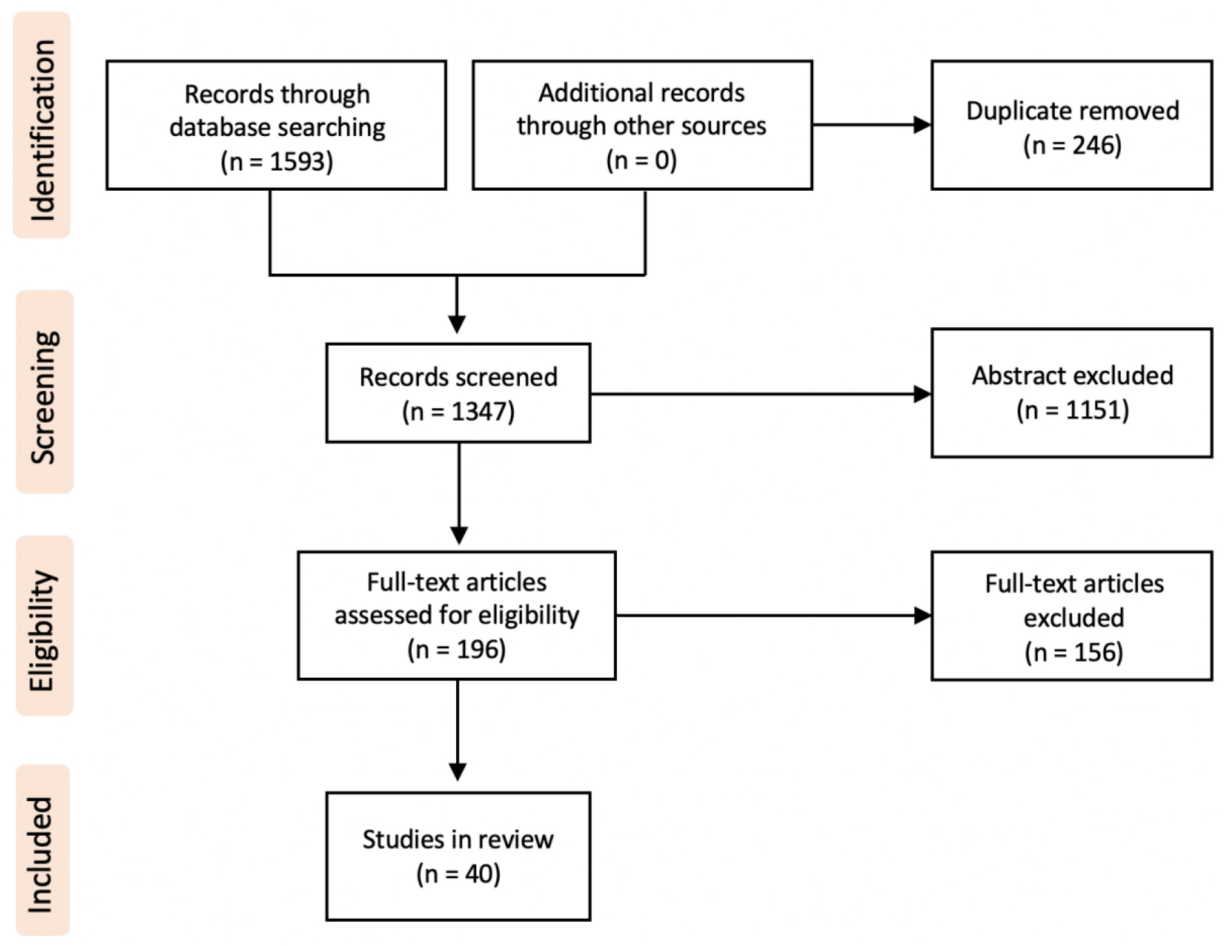
PRISMA chart.

Of 1593 identified articles between 2002 and 2022, 40 fulfilled the review criteria (Figure 1). All these reports had a level of evidence of IV or V. A total of 60 pediatric cases with traumatic and non-traumatic MO were identified (Table 1). The mean age of the patients at diagnosis was 9.5 years (range 0.2–17 years). The male-to-female ratio was 1:1. For further data analysis, the identified cases were respectively separated into an atraumatic group (AGMO) and a traumatic group (TGMO) (Table 2).

**Table 1:** Demographic and clinical characteristics of included cases.

Author	Year	Sex	Age	Location	Trauma	Initial diagnosis	Imagery	Biopsy	Treatment	Follow-up
Yazici et al. (9)	2002	M	8 years	Paravertebral	No	Neoplasia	Rx, US, CT	None	Excision	Clinical
Jayasekera et al. (10)	2005	M	15 years	Hand	Excessive activity	MO	Rx, US, CT, MRI	None	Conservative	Clinical
Chadha et al. (11)	2007	M	10 years	Elbow, wrist	Yes	MO	Rx	UD	Conservative	Clinical
Cabello et al. (12)	2008	M	4 years	Arm	No	Neoplasia	Rx, MRI, BS, PET-CT	Percutaneous	Excision	Clinical
Michelli et al. (6)	2009	M	11 years	Thigh	Yes	Infection	Rx, US, CT, MRI	Incisional	Excision	Rx, CT
Leung et al. (13)	2010	M	11 years	Knee	Yes	UD	MRI	None	Excision	Clinical
Utumi et al. (14)	2010	M	17 years	Cheek	No	Neoplasia	Rx, CT	Incisional	Excision	Rx, CT
Findlay et al. (15)	2010	M	8 years	Neck	Yes	MO	Rx, CT, MRI	None	Conservative	CT
Koob et al. (16)	2010	F	10 years	Chest	Yes	Neoplasia	Rx, CT, BS, PET-CT, MRI	Incisional	Chemoth., conserv.	CT
Davies et al. (17)	2011	F	15 years	Knee	Yes	MO	Rx, US, MRI	None	Conservative	MRI
Jujena et al. (18)	2011	M	6 years	Bilat. tigh	No	Infection	Rx, CT	None	Conservative	Rx
Man et al. (19)	2011	F	17 years	Neck	Yes	Infection	US, CT, MRI	None	Excision	US
Harmon et al. (20)	2012	F	2,5 months	Neck	Yes	Neoplasia	Rx, CT, MRI	Percutaneous	Conservative	Rx
Lau et al. (21)	2012	F	16 years	Thigh	No	Neoplasia	Rx, US, MRI	Percutaneous	Conservative	Clinical
De Smet et al. (22)	2012	F	12 years	Hand	Yes	MO	Rx, US, CT	None	Excision	UD
Mani-Babu et al. (23)	2014	M	14 years	Thigh	Yes	MO	Rx, MRI	None	Conservative	Clinical
Masquijo et al. (24)	2014	F	16 years	Psoas	Excessive activity	Neoplasia	Rx MRI	Percutaneous	Conservative	MRI
Kanthimati et al. (25)	2014	M	13 years	Elbow	Yes	MO	Rx	None	Excision	Rx
Say et al. (26)	2015	F	10 years	Forearm	No	Neoplasia	Rx, MRI	UD	Excision	Clinical
Akahane et al. (27)	2015	F	15 years	Hand	No	UD	Rx, US, MRI	Incisional	Excision	Clinical
Yamaga et al. (28)	2015	F	11 years	Shoulder	No	Neoplasia	CT, MRI, PET-CT	Incisional	Conservative	Rx
Li et al. (29)	2016	M	9 years	Elbow	No	Infection	Rx, CT	Percutaneous	Excision	Clinical
Lin et al. (30)	2016	M	13 years	Knee	No	Neoplasia	Rx, CT	None	Excision (2x)	Rx
Simmonds et al. (31)	2016	F	5 months	Neck	No	UD	MRI	Percutaneous	Excision	Clinical
Becker et al. (32)	2016	M	17 years	Cheek	Yes	Infection	CT	None	Excision	CT
Desai et al. (8)	2017	F	12 years	Popliteal	Excessive activity	MO	Rx, MRI	None	Excision	Rx
Sferopoulos et al. (5)	2017	F (11) M (9)	3–14 years	Pelvis (6) Thigh (2) Elbow (10) Chest (1) Hip (1)	No (1) Yes (19)	UD (20)	Rx (20)	None	Excision (4) Conservative (16)	Rx (1) UD (19)
Mohamed et al. (33)	2018	M	15 years	Hip	No	MO	Rx	None	Excision	Clinical
Kougias et al. (34)	2019	F	5 years	Bilat. hip	No	MO	Rx, CT	None	Conservative	Rx
		M	5 years	Bilat. hip	No	MO	Rx, CT, BS	None	Conservative	Rx, CT
Onen et al. (35)	2019	F	5 years	Lumbar	No	MO	Rx, CT, MRI	UD	Excision	Rx
Dubuisson et al. (36)	2019	M	5 years	Neck	No	Neoplasia	Rx, US, CT, MRI, BS	Incisional	Excision	MRI
Palla et al. (37)	2020	F	7 years	Jaw	Yes	MO	CT	None	Excision	Clinical
Akatli et al. (38)	2021	F	13 years	Pectoralis	No	Infection	US, MRI	None	Excision	Clinical
Chen et al. (39)	2021	M	9 years	Elbow	Yes	MO	Rx, US, CT, MRI	None	Excision	Rx
Rehman et al. (40)	2021	F	12 years	Axilla	No	Infection	US, MRI	None	Excision	Clinical
Cao et al. (41)	2021	F	8 years	Elbow	Yes	MO	Rx	None	Conservative	Rx
Dennison et al. (42)	2022	M	At birth	Leg	No	Neoplasia	Rx, CT, MRI	Percutaneous	Conservative	Rx
Vitale et al. (43)	2022	M	14 years	Neck	No	Neoplasia	Rx, US, CT, MRI	Percutaneous	Excision	UD
Xia et al. (44)	2022	F	8 years	Thigh	No	Infection	Rx, US, CT	Incisional	Excision	Clinical
Silveri et al. (45)	2022	M	2 years	Elbow	No	MO	Rx, MRI, BS	Percutaneous	Conservative	MRI

UD, undocumented; Y, year(s); M, month(s); Rx, Radiography; US, Ultrasound; CT, Computed Tomography; MRI, Magnetic Resonance Imaging; BS, Bone Scintigraphy

**Table 2:** Clinical and management differences between in the TGMO and AGMO.

	All cases of MO *n* = 60	TGMO *n* = 37	AGMO *n* = 23	*P*
Sex				
Male	30	17 (45.9)	13 (56.5)	0.42
Female	30	20 (54.1)	10 (43.5)	0.42
History trauma				
Yes	34	34 (91.9)	0	
Activity	3	3 (8.1)	0	
No	23	0	23 (100)	
Initial diagnosis				
MO	16	11 (29.7)	5 (21.7)	0.65
Neoplasia	13	3 (8.1)	10 (43.5)	0.001
Infection	8	3 (8.1)	5 (21.7)	0.13
Undocumented	23	21 (56.7)	3 (13.0)	
Location				
Upper limb	23 (38.3)	16 (43.0)	7 (30.4)	0.32
Lower limb	24 (40)	15 (40.5)	9 (39.1)	0.92
Trunk	4 (8.3)	1 (2.7)	3 (13.0)	0.12
Head and neck	9 (15.0)	5 (13.5)	4 (17.4)	0.68
Imaging				
Radiography	52	32 (86.5)	20 (86.7)	0.96
Ultrasound	14	6 (16.2)	8 (34.8)	0.099
CT	23	10 (27.0)	13 (56.5)	0.009
MRI	25	11 (29.7)	14 (60.9)	0.017
PET	3	1 (2.7)	2 (8.7)	0.30
Bone scintig.	5	1 (2.7)	4 (17.4)	0.045
Biopsy				
None	41	32 (86.5)	9 (39.1)	0.0001
Percutaneous	9	2 (5.4)	7 (30.4)	0.008
Incisional	7	2 (5.4)	5 (21.7)	0.02
Undocumented	3	1 (2.7)	2 (8.7)	
Treatment				
Conservative	32	25 (67.6)	7 (30.4)	0.01
Surgical	28	13 (35.1)	15 (65.2)	0.01
Follow-up				
Clinical	16	5 (13.5)	11 (47.8)	0.01
Radiological	23	12 (32.4)	11 (47.8)	0.39
Undocumented	21	20 (54.1)	1 (4.3)	

Twenty-three patients (38.3%) were diagnosed with idiopathic/pseudomalignant MO (=AGMO) and 37 patients (61.7%) with circumscribed MO (=TGMO). In the TGMO, a traumatic triggering event was recorded in 34/37 cases, while minor repetitive trauma or excessive activity was incriminated in 3/37 cases. All cases of AGMO had no triggering trauma identified upon clinical history.

In general, MO was localized in the muscle groups of the upper limb (23), the lower limb (13), the pelvis (11), the neck (6), the head (3), the chest wall (2), and the back (2). We observed a similar anatomical distribution between the AGMO and TGMO groups.

During the diagnosis process, the imaging investigations performed were 52 radiographs (86.6%), 14 ultrasounds (23.3%), 23 CTs scans (38.3%), 25 MRIs (41.6%), 5 bone scintigraphies (8.3%), and 3 PETs (5%). Based on clinical and imaging findings, the initial pre-emptive diagnosis was MO in 16/60 patients (26.7%), infection in 8/60 patients (13.3%), and neoplasia in 13/60 patients (21.7%). The remaining cases did not document this information.

Tissue samples were obtained by achieving percutaneous biopsy in 9 patients (15%), incisional biopsy in 7 patients (11.7%), and straightforward resection in 19 patients (31.7%). Biopsy was repeated in one patient due to a non-conclusive first histopathological examination. Four cases in the TGMO (10.8%) against 12 cases in the AGMO (52,2%) needed a biopsy to achieve a diagnosis.

First-line treatment was non-surgical in 32 patients (53.3%). Conservative treatment includes the use of anti-inflammatory drugs, corticosteroids, rest, or physiotherapy. Bisphosphonate injections were performed in only one case. Four children who failed conservative treatment had subsequent surgical excision. Surgical excision was the first-line treatment in 28 patients (46.7%). Detailed treatment strategies within the AGMO and TGMO subgroups are illustrated in Table 2.

The follow-up strategy was clinical in 16 patients (26.7%) or based on imaging investigation in 23 patients (38.3%) and was not documented in 21 cases (35%). The type of imaging performed for follow-up was radiography alone (20%), MRI (6.7%), CT scan alone (5%), radiography and CT combined (5%), and ultrasound (1.6%). Recurrence occurred in one case supposedly because of early surgical excision.

## Discussion

Pseudo-malignant and circumscribed MO is a benign and self-limiting ossification within the soft tissue. The typical evolution of MO is the growth of a firm extraskeletal mass for several months until reaching its peak dimension and then spontaneously resolving (20). The exact causes of MO are not well understood. It may be related to a single and direct trauma, recurrent minor trauma, microscopic damage due to muscle overuse, orthopedic or maxillofacial operations, or muscular bleeding in hemophilic children (24, 32, 46).

### Demographics

MO in the pediatric population can occur at any age with a mean of 9.5 years old (range 0.2–17 years). In the general population, it typically involves young adults with a mean age of 36 years old (range 4–84 years). The sex ratio was balanced in our review (30♀/30♂) while previous reviews performed on the general population showed a significant masculine tendency (7).

### Clinical presentation

As observed in the general population, MO is mostly located in the large skeletal muscle of the extremities (76.6%). The upper and lower extremity have shown the same prevalence of 38.3% and 40% respectively. The head and neck, and the trunk are less frequent with a prevalence of 15.0% and 8.3% respectively.

Myosistis ossificans can clinically mimic extra-skeletal osteosarcoma, parosteal osteosarcoma, rhabdomyosarcoma, synovial sarcoma, and lymphoma. In this review, neoplasia was the initial pre-emptive diagnosis in at least 21.7% of the cases, illustrating an appreciable level of suspicion for this differential. We have also observed an increased level of suspicion for neoplasia when a history of trauma is absent (*p* = 0.001). This is, we believe, why clinicians are driven to perform further imaging and histopathological investigations (47).

### Imaging

**Figure 2:**
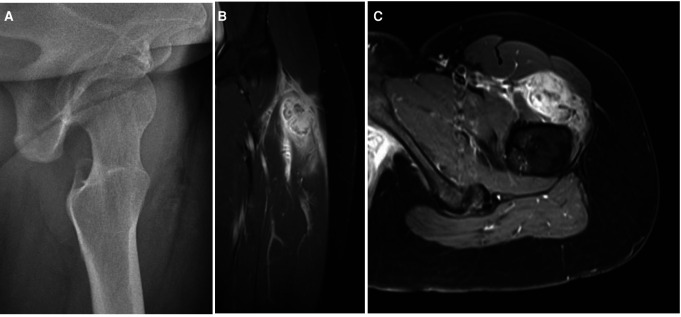
(**A**) Radiograph and (**B,C**) MRI finding in an adolescent with a circumscribed myositis ossificans lesion on the left thigh, one month after the triggering event.

Imaging findings change through the evolution of MO. At the onset, radiography is usually negative or may assess soft tissue swelling. In the presence of trauma, soft tissue swelling seen in early radiography is often mistaken for intramuscular hemorrhage (Figure 2A). Three to four weeks after the triggering event, a peripheral pattern of calcification with a low-density center starts to appear (48). A similar evolution over time is observed in computed tomography and MRI (Figure 2B). Pathognomonic MRI features of advanced MO associate a well-defined heterogeneous mass with a signal intensity approaching bone marrow enhanced by contrast injection, and a rim of cortical calcification independent of the underlying bone (49). MRI may show prominent lymph nodes, signaling inflammatory response, and may be confused with a metastatic process (50). This hyperinflammatory state is also sensitively appreciated with an intake signal on PET CT and 99mTc diphosphonate bone scintigraphy (12, 28). Features in early imaging are not pathognomonic and therefore create confusion. This initial phase of uncertainty seems to drive clinicians to perform further imaging. Indeed, we have assessed a significant difference in the need for MRI and CT imaging, in favor of the AGMO, suggesting a more difficult diagnostic reflection when no triggering trauma is documented.

### Biopsy and histological features

**Figure 3:**
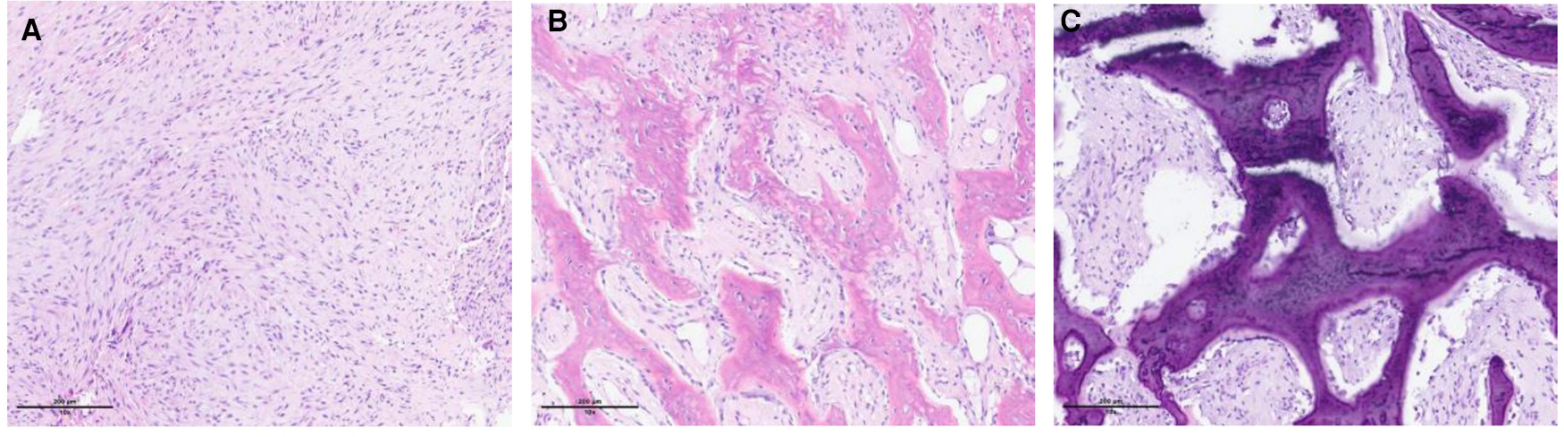
Microscopic illustration of the zonal phenomenon. (**A**) Central zone (proliferation of fibroblastic cells interspersed with macrophages). (**B**) Intermediate zone (osteoblasts, immature bone islands). (**C**) Peripheral zone (mature lamellar bone).

The typical microscopic findings observed in matured MO are described as the zonal phenomenon (Figure 3) (5). The tumor presents a progressive maturation evolving in four levels from its center to its periphery. At the core, multiple immature and proliferative cells are packed. In the adjacent zone, cellular osteoids are observed and separated by a cellular stroma. In the more peripheral zone, osteoblasts and fibrous tissue are organized following a trabecular pattern. The outer layer is composed of matured bone and a fibrous capsule. Excisional and open biopsies procure sufficient tissue material to highlight the zonal phenomenon. Therefore, a fine-needle biopsy may fail to procure enough specimens to demonstrate the zonal phenomenon. A single specimen taken from the center of the lesion may be confused with the findings of a malignant process.

We noted the case of a 10-year-old boy with a chest mass mistakenly diagnosed as osteosarcoma on histology and therefore treated with chemotherapy. The authors of this report declare to have performed a biopsy too early in the clinical evolution leading to confusion. Conclusively, a biopsy performed in the early stage of evolution provides no specific findings, may worsen pain by exacerbating local inflammation, and can lead to diagnostic errors and subsequently to iatrogenic harm. Therefore, the timing of the biopsy should be carefully planned to perform adequate diagnosis.

In the absence of triggering trauma, a significantly greater need for biopsy was observed in this review. This sheds light on the importance of a meticulous history looking for a history of trauma or a cause of muscular micro-injuries (e.g., excessive activity). When confronted with MO in infants, the battered child syndrome must be raised and evaluated, especially in supposed non-traumatic MO.

In addition to histological features consistent with MO, two authors have assessed the presence of COL1A1-USP6 transcript at PCR analysis (38, 43). These were not described previously in the pediatric population. Reports on adult cases have already noticed USP6 gene rearrangement in MO (32, 51). The potential correlation between this genetic alteration and MO should be investigated by larger sample studies.

### Treatment

Historically, surgical excision during the early inflammatory phase led to recurrence and confusion upon pathology analysis. Early supportive treatment is key, by combining anti-inflammatory drugs and muscle rest to calm soft tissue edema. Surgical excision on matured lesions can be proposed usually between the 6th and 12th months if confronted with persistent pain despite adequate analgesic, significant mobility restriction especially trismus, vascular compression, or threat to noble anatomical structure such as respiratory tract in neck involvements. Local compression and intense physiotherapy should follow surgical excision if performed (32). A tendency for non-operative treatment is observed in the PGMO (*p* = 0.01), whereas more surgical excisions were observed in the AGMO.

### Follow-up

We assessed that the follow-up strategy is not well defined. Authors have performed clinical follow-up as well as radiological follow-up in an even distribution. Ultrasonography, radiography, MRI, or even CT were the modality used in the radiological follow-up strategy. The question of unnecessary irradiation and cost should be raised in a disease known to be self-resolving. Moreover, Becker and colleagues observed the persistence of calcification on CT scan in a clinically asymptomatic patient (32). Except for MO located in compromising anatomical sites, clinical follow-up with the clinical assessment of pain resolution, and mobility recovery is sufficient. Even though MO is considered a benign pathology, local recurrences following serial surgical excision have been described in a 10 years-old boy by Lin and colleagues (30). True recurrence rate could not be assessed due to the short follow-up period (less than 12 months) in most reports.

## Conclusion

Over the last 20 years, 60 cases of MO of which 37 circumscribed and 23 pseudo-malignant were reported in the literature, confirming the rarity of the pathology in the pediatric population. This review emphasizes the importance of a meticulous history when MO is suspected. Indeed, clinicians should acknowledge that the absence of a triggering trauma tends to direct the investigation and the management towards a surgical attitude. Furthermore, the timing of the biopsy and radiography should be carefully planned to perform to avoid diagnosis confusion and iatrogenic harm. Paradoxically, in a condition known to be self-limited, a straightforward resection was achieved in nearly one-third of cases. Conservative management is key and surgical excision may be proposed on matured lesions on a case-by-case basis. There is no consensus regarding the need for radiography upon follow-up. However, clinicians should be aware that despite the benignity of MO recurrence has been documented.

## Data Availability

The datasets presented in this study can be found in online repositories. The names of the repository/repositories and accession number(s) can be found in the article/Supplementary Material.

## References

[1] Walczak BE Johnson CN Howe BM. Myositis ossificans. J Am Acad Orthop Surg. (2015) 23(10):612–22. 10.5435/JAAOS-D-14-00269 26320160

[2] Kan L Kessler JA. Evaluation of the cellular origins of heterotopic ossification. Orthopedics. (2014) 37(5):329–40. 10.3928/01477447-20140430-07 24810815

[3] Kaplan FS Glaser DL Hebela N Shore EM. Heterotopic ossification. J Am Acad Orthop Surg. (2004) 12(2):116–25. 10.5435/00124635-200403000-00007 15089085

[4] Mavrogenis AF Soucacos PN Papagelopoulos PJ. Heterotopic ossification revisited. Orthopedics. (2011) 34(3):177. 10.3928/01477447-20110124-08 21410128

[5] Sferopoulos NK Kotakidou R Petropoulos AS. Myositis ossificans in children: a review. Eur J Orthop Surg Traumatol. (2017) 27(4):491–502. 10.1007/s00590-017-1932-x 28275867

[6] Micheli A Trapani S Brizzi I Campanacci D Resti M de Martino M. Myositis ossificans circumscripta: a paediatric case and review of the literature. Eur J Pediatr. (2009) 168(5):523–9. 10.1007/s00431-008-0906-8 19130083

[7] Saad A Azzopardi C Patel A Davies AM Botchu R. Myositis ossificans revisited – the largest reported case series. J Clin Orthop Trauma. (2021) 17:123–7. 10.1016/j.jcot.2021.03.005 33816108 PMC7995649

[8] Desai VS Kakazu R Crawford AH Stanek JW. An unusual presentation of myositis ossificans in a pediatric patient. J Pediatr Orthop. (2017) 37(1):e48–52. 10.1097/BPO.0000000000000669 26491916

[9] Yazici M Etensel B Gürsoy MH Aydoğdu A Erkuş M. Nontraumatic myositis ossificans with an unusual location: case report. J Pediatr Surg. (2002) 37(11):1621–2. 10.1053/jpsu.2002.36196 12407551

[10] Jayasekera N Joshy S Newman-Sanders A. Myositis ossificans traumatica of the thenar region. J Hand Surg Br. (2005) 30(5):507–8. 10.1016/j.jhsb.2005.06.011 16084631

[11] Chadha M Agarwal A. Myositis ossificans traumatica of the hand. Can J Surg. (2007) 50(6):E21–22. 18067695 PMC2386223

[12] Cabello García D Rodríguez Fernández A Gómez Río M Moreno MJ Rebollo Aguirre AC Martín Castro A Circumscript myositis ossificans in a four-year-old boy. Rev Esp Med Nucl. (2008) 27(5):358–62. 10.1157/13126193 18817666

[13] Leung AH Rybak LD Rose DJ Desai P. Myositis ossificans within the intercondylar notch treated arthroscopically. Skeletal Radiol. (2010) 39(9):927–30. 10.1007/s00256-010-0928-y 20532499

[14] Utumi ER Pedron IG Zambon CE Neto NPC Rocha AC. Rare occurrence of myositis ossificans traumatica in a patient with rubinstein-taybi syndrome. J Oral Maxillofac Surg. (2010) 68(10):2616–22. 10.1016/j.joms.2009.08.030 20863946

[15] Findlay I Lakkireddi PR Gangone R Marsh G. A case of myositis ossificans in the upper cervical spine of a young child. Spine. (2010) 35(25):E1525. 10.1097/BRS.0b013e3181ec066b 21102285

[16] Koob M Durckel J Dosch JC Entz-Werle N Dietemann JL. Intercostal myositis ossificans misdiagnosed as osteosarcoma in a 10-year-old child. Pediatr Radiol. (2010) 40(1):34–7. 10.1007/s00247-010-1769-5 20614112

[17] Davies JF Chandramohan M Groves C Grogan RJ Bollen S. Myositis ossificans as a complication of hamstring autograft harvest for open primary anterior and posterior cruciate ligament and posterolateral corner reconstruction. Knee Surg Sports Traumatol Arthrosc. (2011) 19(1):108–11. 10.1007/s00167-010-1184-3 20552160

[18] Juneja M Jain R Mishra D Gautam VK. Myositis ossificans of bilateral hip joints in a patient with diplegic cerebral palsy. J Clin Neurosci. (2011) 18(4):580–1. 10.1016/j.jocn.2010.07.143 21316244

[19] Man SC Schnell CN Fufezan O Mihut G. Myositis ossificans traumatica of the neck – a pediatric case. Maedica (Bucur). (2011) 6(2):128–31. 22205895 PMC3239391

[20] Harmon J Rabe AJ Nichol KK Shiels WE. Precervical myositis ossificans in an infant secondary to child abuse. Pediatr Radiol. (2012) 42(7):881–5. 10.1007/s00247-011-2270-5 22037930

[21] Lau J Hartin CWJ Ozgediz DE. Myositis ossificans requires multiple diagnostic modalities. J Pediatr Surg. (2012) 47(9):1763–6. 10.1016/j.jpedsurg.2012.05.009 22974621

[22] De Smet L Degreef I. Myositis ossificans of the hand in a child: case report. J Pediatr Orthop B. (2012) 21(6):539. 10.1097/BPB.0b013e3283524bfa 22568961

[23] Mani-Babu S Wolman R Keen R. Quadriceps traumatic myositis ossificans in a football player: management with intravenous pamidronate. Clin J Sport Med. (2014) 24(5):e56–58. 10.1097/JSM.0000000000000034 24184852

[24] Masquijo JJ Sartori F. Myositis ossificans circumscripta of the psoas muscle due to overuse in an adolescent gymnast. J Pediatr Orthop B. (2014) 23(6):529–32. 10.1097/BPB.0000000000000096 25171567

[25] Kanthimathi B Udhaya Shankar S Arun Kumar K Narayanan VL. Myositis ossificans traumatica causing ankylosis of the elbow. Clin Orthop Surg. (2014) 6(4):480–3. 10.4055/cios.2014.6.4.480 25436075 PMC4233230

[26] Say F Coskun S Bülbül M Alici Ö. Myositis ossificans on the forearm in a 10-year-old girl. J Pediatr Orthop B. (2015) 24(3):223. 10.1097/BPB.0000000000000152 25647566

[27] Akahane T Mori N Nakatsuchi Y. Myositis ossificans occupying the thenar region: a case report. J Med Case Rep. (2015) 9:105. 10.1186/s13256-015-0586-8 25943356 PMC4428242

[28] Yamaga K Kobayashi E Kubota D Setsu N Tanaka Y Minami Y Pediatric myositis ossificans mimicking osteosarcoma. Pediatr Int. (2015) 57(5):996–9. 10.1111/ped.12672 26508182

[29] Li PF Lin ZL Pang ZH. Non-traumatic myositis ossificans circumscripta at elbow joint in a 9-year old child. Chin J Traumatol. (2016) 19(2):122–4. 10.1016/j.cjtee.2016.01.009 27140223 PMC4897847

[30] Lin RL Liu ZJ Zhang LJ. A rare repeated recurrence of myositis ossificans in the lateral right knee. Int J Clin Exp Med. (2016) 9(6):12212–7.

[31] Simmonds J Taki N Chilton I Vecchiotti M. A rare case of pediatric nontraumatic myositis ossificans in the posterior triangle. Int J Pediatr Otorhinolaryngol. (2016) 84:116–8. 10.1016/j.ijporl.2016.03.003 27063765

[32] Becker OE Avelar RL Rivero ERC De Oliveira RB Meurer MI Santos AM Myositis ossificans of the temporalis muscle. Head Neck Pathol. (2016) 10(3):340–4. 10.1007/s12105-015-0675-4 26703385 PMC4972749

[33] Mohamed AWA Moussa K Seyni SB Seyni ZA Kasoumou AS Ziberou K. Myositis ossificans circumscripta of the hip: about a case. Pan Afr Med J. (2018) 29:207. 10.11604/pamj.2018.29.207.14126 30100961 PMC6080976

[34] Kougias V Hatziagorou E Laliotis N Kyrvasillis F Georgopoulou V Tsanakas J. Two cases of myositis ossificans in children, after prolonged immobilization. J Musculoskelet Neuronal Interact. (2019) 19(1):118–22. 30839310 PMC6454263

[35] Onen MR Varol E Mİ T Naderi S. Nontraumatic myositis ossificans as an uncommon cause of scoliosis: case report and review of the literature. World Neurosurg. (2019) 123:208–11. 10.1016/j.wneu.2018.11.259 30576826

[36] Dubuisson A Lombard A Otto B. Pseudomalignant myositis ossificans of the neck in a child: case report and review of the literature. World Neurosurg. (2019) 130:95–7. 10.1016/j.wneu.2019.06.165 31260851

[37] Palla B Han MD Callahan N. Myositis ossificans traumatica of the head and neck in a child. J Dent Child. (2020) 87(2):120–3. 32788007

[38] Akatli AN Uguralp S Alan S Tasci A Yildirim G. Solitary bone cyst like areas in myositis ossificans: a breast mass in a child. Fetal Pediatr Pathol. (2021) 40(3):262–70. 10.1080/15513815.2019.1693673 31757181

[39] Chen J Li Q Liu T Jia G Wang E. Bridging myositis ossificans after supracondylar humeral fracture in a child: a case report. Front Pediatr. (2021) 9:746133. 10.3389/fped.2021.746133 34869103 PMC8636899

[40] Rehman N Sadashiva H Madakshira MG Raman DK. Non-traumatic myositis ossificans. Autops Case Rep. (2021) 11:e2021316. 10.4322/acr.2021.316 34458184 PMC8387081

[41] Cao J Zheng HJ Sun JH Zhu HY Gao C. Case report: unusual presentation of myositis ossificans of the elbow in a child who underwent excessive postoperative rehabilitation exercise. Front Pediatr. (2021) 9:757147. 10.3389/fped.2021.757147 34869112 PMC8633484

[42] Dennison CB Royall IR Beavers KM Dean CW Scherer KF. Myositis ossificans: a rare neonatal presentation. Pediatr Radiol. (2022) 52(3):587–91. 10.1007/s00247-021-05204-7 34601621

[43] Vitale V Bleve C Mansour M De Corti F Giarraputo L Brugiolo A Non-traumatic myositis ossificans as unusual cause of neck pain during COVID-19 pandemic: a case report. SN Compr Clin Med. (2022) 4(1):96. 10.1007/s42399-022-01177-2 35434525 PMC9005316

[44] Xia AN Wang JS. Giant nontraumatic myositis ossificans in a child: a case report. World J Clin Cases. (2022) 10(9):2901–7. 10.12998/wjcc.v10.i9.2901 35434084 PMC8968807

[45] Silveri C Stoppiello P Gaiero L Bianchi G Casales N Belzarena AC. Aggressive atraumatic myositis ossificans in a toddler. Radiol Case Rep. (2022) 17(12):4550–5. 10.1016/j.radcr.2022.09.032 36193266 PMC9526017

[46] Gindele A Schwamborn D Tsironis K Benz-Bohm G. Myositis ossificans traumatica in young children: report of three cases and review of the literature. Pediatr Radiol. (2000) 30(7):451–9. 10.1007/s002479900168 10929363

[47] Ben-Youssef L Schmidt TL. Battered child syndrome simulating myositis. J Pediatr Orthop. (1983) 3(3):392–5. 10.1097/01241398-198307000-00023 6874942

[48] McCarthy EF Sundaram M. Heterotopic ossification: a review. Skeletal Radiol. (2005) 34(10):609–19. 10.1007/s00256-005-0958-z 16132978

[49] Tyler P Saifuddin A. The imaging of myositis ossificans. Semin Musculoskelet Radiol. (2010) 14(2):201–16. 10.1055/s-0030-1253161 20486028

[50] Kokkosis AA Balsam D Lee TK Schreiber ZJ. Pediatric nontraumatic myositis ossificans of the neck. Pediatr Radiol. (2009) 39(4):409–12. 10.1007/s00247-009-1165-1 19229531

[51] Flucke U Bekers EM Creytens D van Gorp JM. COL1A1 is a fusionpartner of USP6 in myositis ossificans – FISH analysis of six cases. Ann Diagn Pathol. (2018) 36:61–2. 10.1016/j.anndiagpath.2018.06.009 29980413

